# Treatment of hemolytic uremic syndrome related to *Bordetella pertussis* infection —is plasma exchange or eculizumab use necessary?

**DOI:** 10.1186/s12882-018-1168-y

**Published:** 2018-12-17

**Authors:** Ken Saida, Masao Ogura, Yuji Kano, Shingo Ishimori, Takahisa Yoshikawa, Hiroko Nagata, Mai Sato, Koichi Kamei, Kenji Ishikura

**Affiliations:** 1 Division of Nephrology and Rheumatology, National Center for Child Health and Development, 2-10-1 Okura, Setagaya-ku, Tokyo, 157-8535 Japan; 2 Department of Pediatrics, Kakogawa Central City Hospital, Hyogo, Japan

**Keywords:** Atypical hemolytic uremic syndrome, *Bordetella pertussis*, Eculizumab, Plasma exchange

## Abstract

**Background:**

*Bordetella pertussis* infection is a known trigger of atypical hemolytic uremic syndrome (HUS). For patients suspected of having atypical HUS, prompt plasma exchange/infusion (PE/PI) or eculizumab (ECZ) treatment is recommended.

**Case presentation:**

We report a 1-month-old female infant who was admitted with a severe cough and a *B. pertussis*-positive sputum culture. She was born at 38 weeks gestation and did not have a family history of renal diseases. Hemophagocytic syndrome was suspected and she was transferred to our hospital 17 days after her initial admission. One day later, she developed acute kidney injury and was diagnosed with HUS triggered by *B. pertussis* infection. Her plasma complement levels were low and her kidney function continued to worsen over the next few days. However, prior to starting ECZ treatment, her kidney function improved spontaneously; she did not receive PE/PI or ECZ. She was discharged 46 days after her initial hospitalization, without complications. A genetic workup revealed no mutations in *CFH*, *CFI*, *CFB, C3*, *MCP*, *THBD*, or *DGKE*.

**Conclusions:**

This case demonstrates that *B. pertussis* infection-related HUS may resolve spontaneously. The decision to treat during the acute phase is challenging because *B. pertussis* often affects infants suspected of having atypical HUS. However, ECZ may not be the first treatment option for patients with *B. pertussis* infection-related HUS unless they show an indicated genetic abnormality; if ECZ is used, early discontinuation should be considered.

## Background

Thrombotic microangiopathy (TMA) includes clinical conditions that present as microangiopathic hemolytic anemia, thrombocytopenia, and organ injury [1]. Generally, TMA syndromes are extraordinarily diverse and may include thrombocytopenic purpura, Shiga toxin-mediated hemolytic uremic syndrome (HUS), complement-mediated HUS (also known as atypical HUS [aHUS]), and other manifestations secondary to an infection, drug, or underlying disease.

Pertussis infection has been a known trigger of aHUS since the initial report of fatal HUS following a pertussis infection in a patient with a suspected factor H mutation [2, 3]. Therefore, in patients with HUS secondary to a *Bordetella pertussis* infection, plasma exchange/infusion (PE/PI) was conducted in most cases during the acute phase because of the possibility of aHUS [4]. Recently, when aHUS has been clinically diagnosed in children, especially in infants, the administration of eculizumab (ECZ), rather than PE/PI, has been considered the first-line treatment [5, 6].

Herein, we report the case of a 1-month-old female infant, with a *B. pertussis* infection, who developed HUS. To our knowledge, this is the first report of a patient whose symptoms resolved, without any complications, and who remained in remission without receiving plasma therapy or ECZ treatment.

## Case presentation

**Fig. 1:**
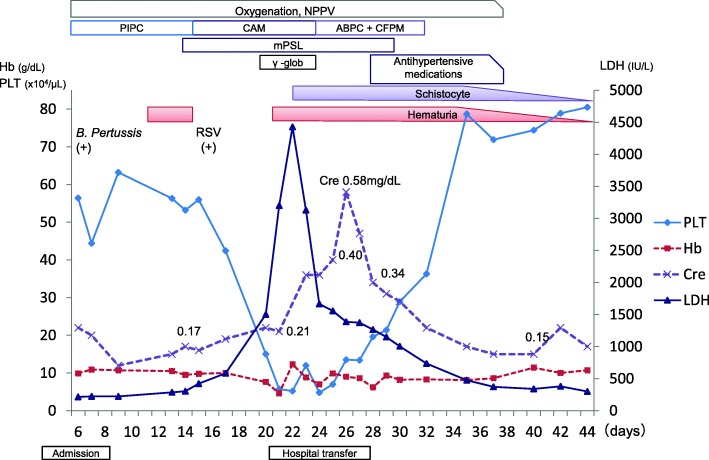
Clinical course of the patient. Platelet counts increased and lactate dehydrogenase and creatinine levels decreased without treatment involving plasma exchange or eculizumab administration NPPV: noninvasive positive pressure ventilation, PIPC: piperacillin, CAM: Clarithromycin, ABPC: Ampicillin, CFPM: Cefepime, mPSL: methylprednisolone, RSV: Respiratory syncytial virus, PLT: Platelets, Hb: Hemoglobin, Cre: Creatinine, LDH: lactate dehydrogenase.

A 1-month-old Japanese girl, born at 38 weeks gestation with a normal birth weight (2870 g) and no family history of TMA or kidney disease, was examined at a hospital due to a 2-day history of cough. She was admitted 3 days later because *B. pertussis* was detected in her nasopharyngeal culture. She was treated with oxygen supplementation, antibiotics (piperacillin), and bronchodilators; her bacterial infection was complicated by a respiratory syncytial virus (RSV) superinfection. Fourteen days after admission, her laboratory evaluation revealed anemia, thrombocytopenia, elevated lactate dehydrogenase (LDH) levels (up to 4,428 IU/L), and markedly increased serum ferritin concentrations (up to 26,208 ng/mL) (Fig. 1). Hemophagocytic syndrome (HPS) was suspected, and treated with steroids and gamma globulin.

She was transferred to our hospital 17 days after her initial admission, and the HPS diagnosis was excluded following a bone marrow analysis. The patient’s plasma complement levels were low (C3, 59 mg/dL; C4, 11 mg/dL; CH50, 31.0 U/mL) and a urinalysis showed hematuria and proteinuria; her kidney function worsened over the next few days (creatinine, up to 0.58 mg/dL). Her ADAMTS13 level was normal, but her haptoglobin level was significantly below normal and schistocytes were found in a peripheral blood smear. As a result, we diagnosed her with HUS caused by *B. pertussis* infection.

During our preparation to initiate ECZ treatment, her LDH levels started decreasing. Thereafter, her creatinine level decreased and her condition improved spontaneously. Hence, we did not perform PE/PI or administer ECZ. The C3 level increased to within normal limits (115 mg/dL). She was discharged 46 days after her first hospitalization, without any complications, and remained in remission 3 years later. A genetic workup was performed to examine for potential complement regulator mutations; however, no mutation was found in *CFH, CFI, CFB, C3, MCP, THBD,* or *DGKE*.

## Discussion and conclusions

Was our case an aHUS triggered by pertussis infection or a secondary TMA due to pertussis infection? It is very difficult to make a decision; however, we consider that this patient is more likely to have had a secondary TMA rather than an aHUS based on the following reasons. First, the HUS of our patient showed spontaneous remission without PE/PI or ECZ treatment. Second, a specific genetic mutation related to complement regulation was not identified. Third, past reports do not describe recurrent HUS after the first episode and our patient did not experience HUS recurrence within 3 years after achieving remission (Table 1).

**Table 1:** Past reports of HUS related to pertussis infection

	1	2	3	4	5	6	The present case
The Age of Onset	20 days	6 weeks	4 weeks	24 days	2 months	1 month	1 month
Sex	M	F	M	F	M	F	F
Duration of Pertussis infection leading to HUS	6 weeks	16 days	21 days	17 days	12 days	18 days	19 days
Intubation	+	–	+	+	+	+	–
LDH	1200	1950	5259	2642	unknown	3268	4428
Plasma therapy	–	PI	PI	PE	PE	PI, PE	–
Dialysis	PD	HD	–	–	PD	HD	–
ECZ	–	–	–	–	–	+	–
Antibiotics	EM	CTX, EM	AMPC, CTX, CAM	AZM	AZM	PIPC, CTX, CAM	PIPC, CAM
Steroid	+	–	–	–	+	–	+
CH50, C3, C4	Normal	Normal	Normal	–	Normal	Normal	Decreased
Gene mutation	*CFH*	–	–	unknown	–	*THBD*	–
Prognosis	Death	Alive	Alive	Alive	Alive	Alive	Alive
Follow-up (year)	–	2	0.6	unknown	1	2.5	3
Author	Berner R. et al. [3]	Pela I. et al. [11]	Chaturvedi S. et al. [4]	Obando I. et al. [12]	Cohen-Ganelin E. et al. [13]	Ito N. et al. [14]	
Reported year	2002	2006	2010	2012	2012	2014	2018

Abbreviations: *LDH* Lactate dehydrogenase, *ECZ* Eculizumab, *PI* Plasma Infusion, *PE* Plasma Exchange, *PD* Peritoneal

Dialysis, *HD* Hemodialysis, *EM* Erythromycin, *CTX* Cefotaxime, *AMPC* Amoxicillin, *CAM* Clarithromycin,

*AZM* Azithromycin, *PIPC* Piperacillin

*B. pertussis* infection-associated HUS was first reported by Berner et al., who suspected a patient of having a *CFH* mutation; the patient had a fatal outcome [3]. Therefore, treatment with PI/PE or ECZ has been performed for most reported cases with *B. pertussis*-related HUS (Table 1). Hence, we are unsure whether these reported patients survived due to treatment-related benefits or due to spontaneous recovery. To our knowledge, ours is the first reported case of suspected aHUS to show a spontaneous recovery, suggesting that HUS secondary to a pertussis infection is actually a secondary TMA. If all patients with potential aHUS receive PI/PE or ECZ treatment, they would probably recover. However, such treatment may be unnecessary for patients with a secondary TMA. Regardless, according to a recent report, immediate (within 24–48 h) administration of ECZ is recommended, especially for pediatric patients suspected of having aHUS [5]. A definite understanding of the clinical presentation of HUS following a pertussis infection, and the judicious use of ECZ, is necessary to avoid unnecessary treatment.

On the other hand, the contribution of complement system dysregulation cannot be completely ruled out in our patient. Even though genetic mutations were not identified, such genetic mutations remain undetected in 30–40% of patients with aHUS [2]. The spontaneous remission of our patient may be due to the effect of the steroid used to treat respiratory symptoms and suspected HPS, prior to her transfer to our hospital. The patient showed decreased levels of both C3 and C4 in the acute phase. Theoretically, aHUS is characterized by abnormalities in the alternative complement pathway and may be identified by a selective C3 deficiency, with normal C4 levels [9]. Conversely, complement levels in secondary TMA are considered to be variable due to its association with a variety of causative diseases. The low complement levels in this patient did not contradict past reports indicating that *B. pertussis* infections induce activation of the classical complement pathway [7].

The pathogenetic triggers of complement activation include immunologic disorders, genetics, infections, systemic diseases, drug administration, and mixed-cause triggers. In Japan, the revised diagnostic criteria for aHUS developed in 2015 by the Japanese Society of Nephrology and the Japan Pediatric Society excluded secondary TMA from the aHUS definition, according to the international consensus [8–10]. Additionally, this new clinical guideline recommends therapeutic treatments, such as ECZ administration or plasma therapy, for patients with aHUS, but not for those with a secondary TMA [8]. Typically, aHUS can be distinguished from other TMAs. However, this may be challenging in the acute phase of HUS onset because pertussis often affects infants < 3-months-old, before they are eligible for post-natal pertussis vaccination. Our case suggests that some cases of HUS following pertussis infection may be secondary TMAs. Conversely, we cannot completely exclude the contribution of an undetected complement regulator abnormality. Determining the appropriate treatment course during the acute phase remains challenging. Clinicians should plan treatments of their patients according to the clinical courses.

In conclusion, *B. pertussis* infection could be a cause of secondary TMA, not an aHUS. ECZ administration or PE/PI may not always be the first treatment option for pediatric patients with HUS secondary to a *B. pertussis* infection. If such treatments are used, their early discontinuation should also be considered.

## Abbreviations

aHUS: Atypical hemolytic uremic syndrome
CFH: Complement factor H
ECZ: Eculizumab
HPS: Hemophagocytic syndrome
HUS: Hemolytic uremic syndrome
PE: Plasma exchange
PI: Plasma infusion
RSV: Respiratory syncytial virus
TMA: Thrombotic microangiopathy
